# Quantitative Characterization of the Toxicities of Cd-Ni and Cd-Cr Binary Mixtures Using Combination Index Method

**DOI:** 10.1155/2016/4158451

**Published:** 2016-12-01

**Authors:** Lingyun Mo, Minyi Zheng, Meng Qin, Xin Zhang, Jie Liu, Litang Qin, Honghu Zeng, Yanpeng Liang

**Affiliations:** ^1^Guangxi Key Laboratory of Environmental Pollution Control Theory and Technology, Guilin University of Technology, Guilin 541004, China; ^2^Collaborative Innovation Center for Water Pollution Control and Water Safety in Karst Area, Guilin University of Technology, Guilin 541004, China

## Abstract

Direct equipartition ray design was used to construct Cd-Ni and Cd-Cr binary mixtures. Microplate toxicity analysis was used to evaluate the toxicity of individual substance and the Cd-Ni and Cd-Cr mixtures on* Chlorella pyrenoidosa* and* Selenastrum capricornutum*. The interacting toxicity of the mixture was analyzed with concentration addition (CA) model. In addition, combination index method (CI) was proposed and used to quantitatively characterize the toxicity of the binary mixtures of Cd-Ni and Cd-Cr observed in experiment and find the degree of deviation from the predicted outcome of the CA model, that is, the intensity of interacting toxicity. Results indicate that most of the 20 binary mixtures exhibit enhancing and synergistic effect, and only Cd-Cr-R4 and Cd-Cr-R5 mixtures have relatively high antagonistic effects against* C. pyrenoidosa*. Based on confidence interval, CI can compare the intensities of interaction of the mixtures under varying levels of effect. The characterization methods are applicable for analyzing binary mixture with complex interaction.

## 1. Introduction

The evaluation and prediction of combined effect of chemical pollutants in the environment have become an interesting research topic in environmental chemistry [1, 2]. Several research on mixtures indicate that the dependency of concentration ratio and effect level with interaction is a common phenomenon [3–7] that requires a reasonable and easily applicable method to represent the toxic interaction of the mixture so as to ensure the reliability of the evaluation results. At present, two additional reference models, namely, concentration addition (CA) and independence action (IA), are widely used to evaluate mixture toxicity. CA model is applicable for mixtures with similar mode of action, whereas the IA model is applicable for mixtures with different modes of action [1, 8–11]. However, the modes of action of many substances are still unknown, and selecting different standards is still difficult, so CA or IA cannot be selected [12, 13]. Through theoretical research and experimental verification, Chou [14–16] proposed to evaluate the toxicity interaction of mixtures using the combination index (CI) based on 50% effective equation without depending on the mode of action. CI has been widely used in assessing the toxicity of drug combinations [17–19]. In recent years, CI has attracted the attention of scholars of environmental sciences [20–22].

All toxicity tests contain experimental error. Different toxicants or pollutants have different concentration-response features, and concentration-response curve (CRC) fitting also contains error of fitting. All these affect the uncertainty of CI index. This research considers the influence of experimental error/different concentration-effect feature and error of fitting on CI. The toxicity interaction is also analyzed by taking the binary mixture of Cd-Ni and Cd-Cr as examples.

## 2. Materials and Methods

### 2.1. Type of Algae, Key Instruments, and Drugs

*Chlorella pyrenoidosa* and* Selenastrum capricornutum* with serial number FACHB-5 and FACHB-271 are purchased from the FACHB-collection of typical culture collection committee of Chinese Academy of Science. BG11 culture medium, which is placed into manual climatic box, is used for cultivation at 22°C, illumination intensity of 3000 lux, and light-dark cycle of 12 h : 12 h. The algae were collected during logarithmic phase for experiment.

Other materials used were as follows: TECAN infinite 200 microplate reader (Switzerland), illumination incubator with constant temperature and artificial climate (Jintan Yitong Electronics Co. Ltd.), 96-hole transparent polystyrene microplate with flat base (Greiner), YXQ-LS-70A vertical pressure steam sterilization, SW-CF-IFD clean bench, Milli-Q ultrapure water system, Haier drug freezer, and Eppendorf pipette.

CdCl_2_·2.5H_2_O[Cd] (Damas-Beta), Ni(NO_3_)_2_·6H_2_O[Ni] (Guangdong Guanghua Technology Stock Co. Ltd.), and K_2_Cr_2_O_7_[Cr] (Damas-Beta) were also used. The prepared solution of the compounds was stored in a refrigerator at 4°C to prepare for the test. See the basic properties of heavy metal compounds in Table 1.

### 2.2. Experimental Design and Toxicity Test

To explore the toxicity interaction of Cd-Ni and Cd-Cr mixtures within the whole space range of concentration, the concentration design of the binary mixtures was created through direct equipartition ray design [4]. The points of 50% effective concentration (EC_50_) of each component on the 2D coordinate planes of concentration comprising two components of the mixture were selected. The two points of EC_50_ were then connected, and the segment was divided into six equal parts. Five radials were made (R1, R2, R3, R4, and R5) from the origin through the point of division. Appropriate dilution factors were selected and 12 points of concentration on each radial were designed. The concentration of each radial can be seen in Tables 2 and 3.

The toxic effects of individual heavy metal and the Cd-Ni and Cd-Cr mixtures on* C. pyrenoidosa* and* S. capricornutum* were evaluated through microplate toxicity analysis (MTA) [23, 24]. Water was added around the 96-hole microplate. On the plate, all the 60 holes on the 6 × 10 array in the middle are used as test holes in toxicity test of algae. There are 24 holes in columns 2, 6, 7, and 11. A total of 100 *µ*L ultrapure water was added in each hole to act as the blank control group. In the remaining 36 holes, we performed three parallel toxicity tests on 12 pollutants with different concentrations designed based on geometric progression, with total volume of the test solution at 100 *μ*L. Finally, 100 *μ*L algae solution was added at logarithmic phase in each of the 60 test holes for toxicity test to come up with a total volume of 200 *μ*L.

Microplate reader tests the absorbancy of each hole on the microplate, and wave length is set as 682 nm. The inhibition of the pollutant on* C. pyrenoidosa* at different exposure duration was calculated using the following equation:

(1) $$\begin{array}{c} E=1-\frac{\left({OD}_{t,i}-{OD}_{t,0}\right)}{\left({OD}_{0,i}-{OD}_{0,0}\right)}, \end{array}$$

where *E* indicates the inhibition of individual compound or mixture with certain concentration on* C. pyrenoidosa* or* S. capricornutum* at different exposure duration; *i* refers to the point of exposure duration of the pollutant, *i* = 0, 1, 2, 3, and 4 corresponding to the exposure duration of 0 h, 24 h, 36 h, 72 h, and 96 h, respectively; OD_*t*,*i*_ is the OD_682_ value of algae solution in pollutant treatment group at *i*; OD_0,*i*_ is the OD_682_ value of algae solution in blank control group at *i*; OD_*t*,0_ is the OD_682_ value of algae solution in pollutant treatment group at 0; and OD_0,0_ is the OD_682_ value of algae solution in blank control group at 0.

### 2.3. Analysis on Toxicity Interaction of Mixture

The synergism, additive effect, or antagonism quantification of binary mixtures was simulated for CI values using CompuSyn [12, 14–16, 25]. The general equation for *n*-drug combination at *x*% inhibition is shown as follows [12]:

(2) CIx∑j=1nDjDxj=∑j=1nDx1−nDj/∑1nDDmjfaxj/1−faxj1/mj,

where (CI)_*x*_ is the combination index for *n* drugs at *x*% inhibition, CI < 1, CI = 1, and CI > 1 indicate synergism, additive effect, and antagonism, respectively, (*D*_*x*_)_*j*_ is the doses of drug *j* alone that inhibit *x*%, (*D*)_*j*_ is the portion of drug *j* in combination that also inhibits *x*%, (*D*_*x*_)_1−*n*_ is the sum of the dose of *n* drugs that exerts *x*% inhibition in combination, (*D*)/∑_1_^*n*^(*D*) is the proportionality of the dose of each of *n* drugs that exerts *x*% inhibition in combination, and (*D*_*m*_)_*j*_{(*fa*_*x*_)_*j*_/[1 − (*fa*_*x*_)_*j*_]}^1/*m*_*j*_^ is the dose of each drug alone that exerts *x*% inhibition, where *D*_*m*_ is the median-effect dose (antilog of the *x*-intercept of the median-effect plot), *fa*_*x*_ is the fractional inhibition at *x*% inhibition, and *m* is the slope of the median-effect plot, which depicts the shape of the dose-effect curve.

## 3. Results and Discussion

### 3.1. Toxicity of Individual Substance on* C. pyrenoidosa*

**Figure 1:**
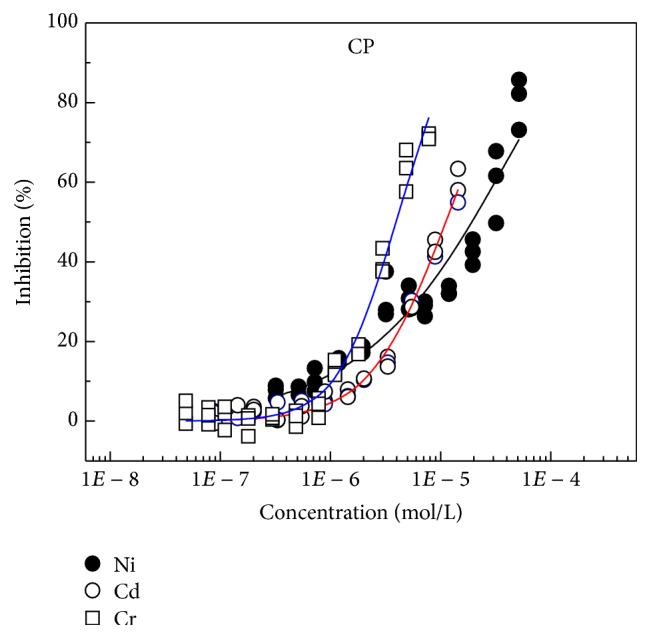
Concentration-response curves of three heavy metals on* C. pyrenoidosa* (CP).

**Figure 2:**
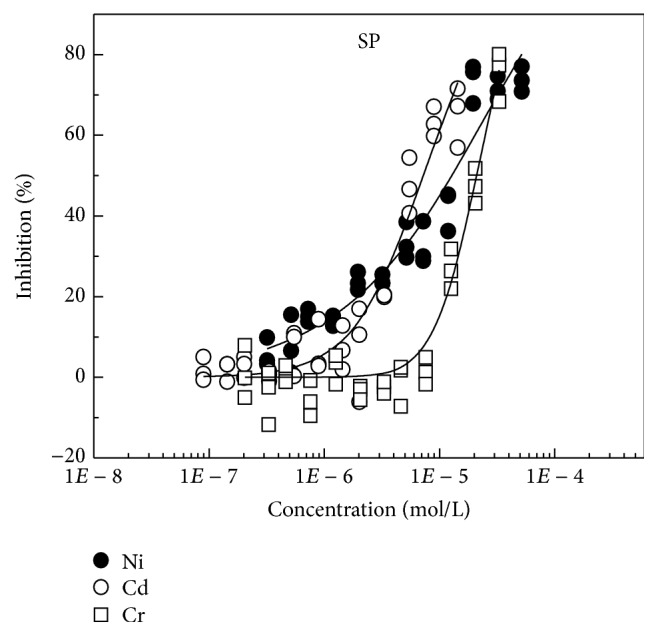
Concentration-response curves of three heavy metals on* S. capricornutum* (SP).

Through MTA, the concentration-response data points (Figures 1 and 2) of Cd, Ni, and Cr on* C. pyrenoidosa* and* S. capricornutum* were tested. The median-effect dose (effect concentration, EC_50_) that inhibits the* pyrenoidosa* and* S. capricornutum* under study by 50% was calculated by CompuSyn software [26]. The *D*_*m*_ (EC_50_) value was calculated by the following:

(3) $$\begin{array}{c} D_{m}=10^{-\left(y\text{-}intercept\right)/m}. \end{array}$$

The order of toxicities of the three heavy metals on* C. pyrenoidosa* is Cr (EC_50_ = 6.11*E* − 6) > Cd (EC_50_ = 1.7*E* − 5) > Ni (EC_50_ = 1.74*E* − 5), and on* S. capricornutum*, the order of toxicities is Cd (EC_50_ = 10.0*E* − 6) > Ni (EC_50_ = 1.15*E* − 5) > Cr (EC_50_ = 6.96*E* − 5). By comparing the toxicities of three heavy metals on* C. pyrenoidosa* and* S. capricornutum*, different green algae were observed to have different tolerance against heavy metals. The tolerance from large to small is Ni (*C. pyrenoidosa* >* S. capricornutum*), Cd (*C. pyrenoidosa* >* S. capricornutum*), and Cr (*S. capricornutum* >* C. pyrenoidosa*). The order of tolerance of* S. capricornutum* and* C. pyrenoidosa* against Cr is consistent with the research results of Chen et al. [27].

### 3.2. Toxicity Analysis of Binary Mixture of Heavy Metal on* C. pyrenoidosa* and* S. capricornutum*

#### 3.2.1. Mixture Characterization

**Figure 3:**
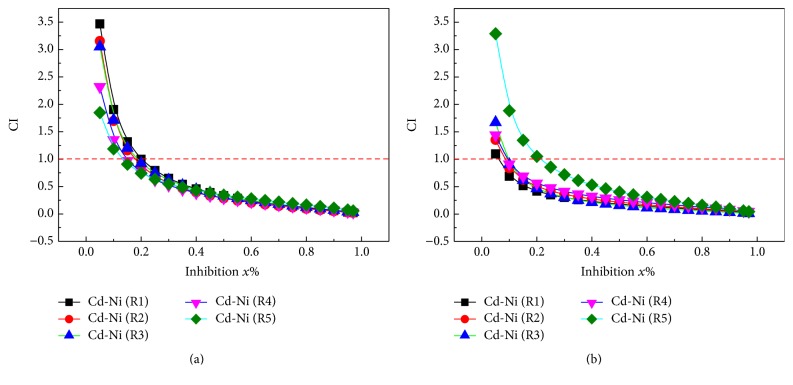
Plot of the combination index versus the effect expressed by Cd-Ni mixed system of binary mixtures. ((a)* C. pyrenoidosa*; (b)* S. capricornutum*).

**Figure 4:**
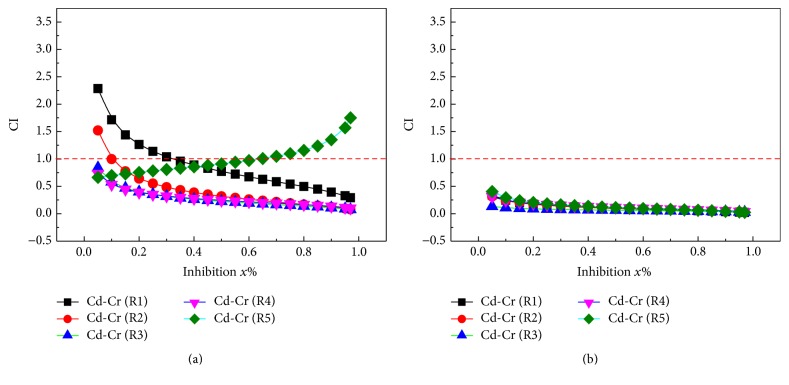
Plot of the combination index versus the effect expressed by Cd-Cr mixed system of binary mixtures. ((a)* C. pyrenoidosa*; (b)* S. capricornutum*).

The CI values of all 20 mixture rays in the binary mixture system for two groups (Cd-Ni and Cd-Cr) were calculated using formula (2). Figures 3 and 4 present the change curve of CI with the effect level (*E*). The concentration ratio of individual in mixtures and EC_50_ for two groups mixtures were listed in Tables 2 and 3.

#### 3.2.2. Cd-Ni Mixture System

Figure 3 indicates the CI of Cd-Ni mixture system on* C. pyrenoidosa* (CP) and* S. capricornutum* (SP). As can be seen from Figure 3(a), the toxicity of Cd-Ni on* C. pyrenoidosa* in low-effect area (lower than 10%) presents an antagonism and synergistic effect in high-effect area (higher than 20%). Mixtures R1–R5 present a synergistic effect in the area with inhibition greater than 30%.

As shown in Figure 3(b), the toxicity of Cd-Ni on* S. capricornutum* in the whole-effect area presents synergism, additive effect, and antagonism. Mixtures R1–R4 present synergistic effect in the area with inhibition greater than 30%, and mixture R5 presents a synergistic effect in the area with inhibition greater than 40%.

In general, in R1–R5 of Cd-Ni mixture, the concentration proportion of Ni decreases with the increase of the concentration proportion of Cd, and the area of synergistic effect had an increasing trend.

#### 3.2.3. Cd-Cr Mixture System

Figure 4 indicates the relationship between the CI and inhibition of Cd-Cr mixture system on* C. pyrenoidosa* and* S. capricornutum* for 96 h.

As seen in Figure 4(a), in Cd-Cr mixture system of R1 toxicity to* C. pyrenoidosa* presents synergistic effect in the high-effect area (higher than 35%), while the effect lower than 35% presents antaganism. Mixture R2 presents synergistic effect in the effect higher than 10% and mixtures R3 and R4 show synergistic effect for the whole effect. Mixture R5 present synergistic effect in the effect lower than 70% and presents antaganism in high-effect area (higher than 70%).

As seen in Figure 4(b), in Cd-Cr mixtures of R1–R5 toxicity to* S. capricornutum* presents synergistic effect in the whole effect. For the toxicity of Cd-Cr system on* S. capricornutum*, Cr concentration gradually increased as the Cd concentration gradually decreased, and the synergistic effect presents in the whole-effect area.

#### 3.2.4. Characterization Results

As seen from Figures 3 and 4, without considering confidence interval, the CI values of radials of many mixtures all deviated from 1 in certain range of effect and interaction exists. For example, the effect of Cd-Ni (R3) on* C. pyrenoidosa* upon 96 h exposure is *x*% = 20–75, that is, synergistic effect, and the effect of Cd-Cr (R2) on* C. pyrenoidosa* 96 h exposure is *x*% = 20–65, that is, antagonism. However, accidental error exists in the toxicity test and error of fitting in nonlinear fitting, so the calculated CI has some degree of uncertainty (indicated by 95% confidence interval in this research).

The results above indicate that, for most mixtures, the same mixture presents synergistic effect or addition action and antagonistic effect on* C. pyrenoidosa* or* S. capricornutum* in the whole effect area. In addition, all the interactions between the five rays of the same mixture system have different changes, mainly because of the difference in mixing ratio. Hence, the concentration proportion of the mixture has greater influence on the type of interaction of the mixtures.

## 4. Conclusions

1. The toxicity interaction of Cd-Ni and Cd-Cr mixtures on* C. pyrenoidosa* or* S. capricornutum* mainly contains addition action and synergistic effect; however, the toxicity effect of Cd-Cr on* C. pyrenoidosa* is addition action in low-effect area. In high-effect area, the effect is antagonistic at effect more than 60%. The concentration range containing antagonism and the intensity of antagonism are affected by several factors, including the components of the mixture, concentration ratio of the components, and the effect level.
2. CI can quantitatively characterize the toxicity effect of the binary mixture from the angle of effect. CI can also compare the intensities of the interaction between the mixtures under any effect level based on confidence interval. The characterization method is applicable for analyzing the binary mixture with complicated interaction.

## Figures and Tables

**Table 1:** Physical properties of three heavy metals.

Compound	CAS	Molecular weight	Purity (%)
CdCl_2_·2.5H_2_O	7790-78-5	228.36	≥98
Ni(NO_3_)_2_·6H_2_O	13478-00-7	290.79	≥98
K_2_Cr_2_O_7_	7778-50-9	294.18	≥99

**Table 2:** Concentration ratio of Cd-Ni and EC_50_ value of 10 binary mixture rays.

Binary mixture	rays	*p*_*i*_		EC_50_
		Cd	Ni	
Cd-Ni toxicity to *C. pyrenoidosa*	R1	0.1034	0.8966	5.77*E* − 6
	R2	0.2239	0.7761	4.94*E* − 6
	R3	0.3658	0.6342	5.73*E* − 6
	R4	0.5357	0.4643	5.08*E* − 6
	R5	0.7425	0.2575	5.96*E* − 6

Cd-Ni toxicity to *S. capricornutum*	R1	0.1009	0.8991	2.04*E* − 6
	R2	0.2192	0.7808	2.39*E* − 6
	R3	0.3595	0.6405	1.72*E* − 6
	R4	0.5289	0.4711	2.75*E* − 6
	R5	0.7373	0.2627	4.16*E* − 6

**Table 3:** Concentration ratio of Cd-Cr and EC_50_ value of 10 binary mixture rays.

Binary mixture	Rays	*p*_*i*_		EC_50_
		Cd	Cr	
Cd-Cr toxicity to *C. pyrenoidosa*	R1	0.9341	0.0659	1.20*E* − 5
	R2	0.8500	0.1500	4.32*E* − 6
	R3	0.7392	0.2608	2.64*E* − 6
	R4	0.5863	0.4137	2.41*E* − 6
	R5	0.3618	0.6382	7.27*E* − 6

Cd-Cr toxicity to *S. capricornutum*	R1	0.0645	0.9355	4.87*E* − 6
	R2	0.1470	0.8530	3.77*E* − 6
	R3	0.2563	0.7437	1.73*E* − 6
	R4	0.4081	0.5919	2.32*E* − 6
	R5	0.6328	0.3672	1.66*E* − 6
